# Demographic reporting and phenotypic exclusion in fNIRS

**DOI:** 10.3389/fnins.2023.1086208

**Published:** 2023-05-09

**Authors:** Jasmine Kwasa, Hannah M. Peterson, Kavon Karrobi, Lietsel Jones, Termara Parker, Nia Nickerson, Sossena Wood

**Affiliations:** ^1^Neuroscience Institute, Carnegie Mellon University, Pittsburgh, PA, United States; ^2^Center for Systems Biology, Massachusetts General Hospital, Boston, MA, United States; ^3^Department of Biomedical Engineering, Boston University, Boston, MA, United States; ^4^Burnett School of Biomedical Sciences, University of Central Florida, Orlando, FL, United States; ^5^Interdepartmental Neuroscience Program, School of Medicine, Yale University, New Haven, CT, United States; ^6^Combined Program in Education and Psychology, University of Michigan, Ann Arbor, MI, United States; ^7^Department of Biomedical Engineering, Carnegie Mellon University, Pittsburgh, PA, United States

**Keywords:** fNIRS (functional near-infrared spectroscopy), inclusion, neuroimaging, melanin, biomedical optics

## Abstract

Functional near-infrared spectroscopy (fNIRS) promises to be a leading non-invasive neuroimaging method due to its portability and low cost. However, concerns are rising over its inclusivity of all skin tones and hair types (Parker and Ricard, 2022, Webb et al., 2022). Functional NIRS relies on direct contact of light-emitting optodes to the scalp, which can be blocked more by longer, darker, and especially curlier hair. Additionally, NIR light can be attenuated by melanin, which is accounted for in neither fNIRS hardware nor analysis methods. Recent work has shown that overlooking these considerations in other modalities like EEG leads to the disproportionate exclusion of individuals with these phenotypes—especially Black people—in both clinical and research literature (Choy, 2020; Bradford et al., 2022; Louis et al., 2023). In this article, we sought to determine if (Jöbsis, 1977) biomedical optics developers and researchers report fNIRS performance variability between skin tones and hair textures, (2a) fNIRS neuroscience practitioners report phenotypic and demographic details in their articles, and thus, (2b) is a similar pattern of participant exclusion found in EEG also present in the fNIRS literature. We present a literature review of top Biomedical Optics and Human Neuroscience journals, showing that demographic and phenotypic reporting is unpopular in both fNIRS development and neuroscience applications. We conclude with a list of recommendations to the fNIRS community including examples of Black researchers addressing these issues head-on, inclusive best practices for fNIRS researchers, and recommendations to funding and regulatory bodies to achieve an inclusive neuroscience enterprise in fNIRS and beyond.

## 1. Introduction

Functional near-infrared spectroscopy (fNIRS) promises to be the leading non-invasive human neuroimaging method of the next few decades due to its portability, low cost, motion tolerance, and usability in special populations. This light-based modality was first ideated for blood-oxygenation estimation and has grown in its popularity, with publication counts doubling every 3.5 years (Jöbsis, 1977; Boas et al., 2014). fNIRS is indispensable in many cognitive and psychological science settings, but especially in child development, hyperscanning, brain-computer interfacing, and other areas where movement and portability are challenges and which preclude EEG and fMRI as the leading non-invasive modalities (Crosson et al., 2010; Yücel et al., 2017; Girolamo et al., 2022).

As fNIRS increases in popularity, concerns over its inclusion of all skin tones and hair types are rising (Parker and Ricard, 2022; Webb et al., 2022). While it has long been established that the physics of hair color, hair thickness, and skin pigmentation affect the detection of a NIRS signal (Pringle et al., 1999), a systematic study is still missing that directly addresses the limitations of modern-day NIRS for different phenotypes. With these limitations, we are in danger of perpetuating bias against the darker skinned and thicker haired people of the world—individuals who already face racism and oppression worldwide. Here, we are careful to distinguish between phenotype and race: while phenotype refers to heritable physical characteristics such as hair and skin color, race is a social construct based on a collection of phenotypic, cultural, and regional indicators that hold power in society and affect the lived experiences of individuals who are minoritized and marginalized based on these indicators.

In this article, we briefly define technical limitations in biomedical optics for marginalized *phenotypes* and explore how they lead to disproportionate exclusion of people of marginalized *races* through a literature review. We sought to examine racial and phenotypic reporting specifically as compared to gender reporting, an established reporting category over the last few decades due to NIH mandated reporting. Although most guidelines combine “women and minorities,” we hypothesized that gender is reported at much higher rates than racial/ethnic demographics and treat it is a reporting exemplar.

## 2. Bias in fNIRS

### 2.1. Phenotypic bias

**Figure 1:**
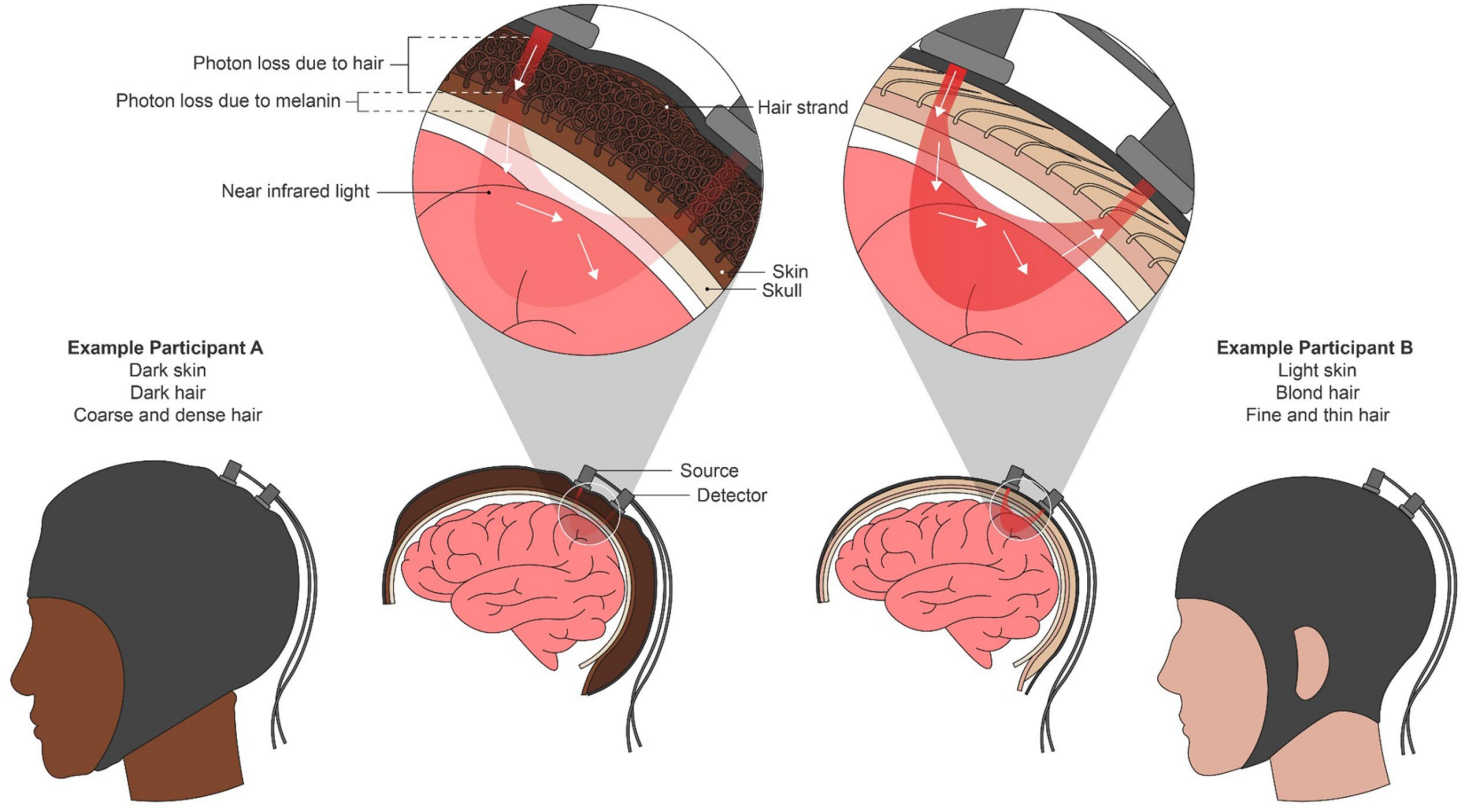
A combination of red and near-infrared (NIR) light at an optical source is shone into the brain non-invasively. From the source, light travels through the skin and into the brain surface before resurfacing at a detector or array of detectors elsewhere on the scalp. Using the scattering and absorption properties of NIR light in brain tissue, the relative amounts of oxygenated and deoxygenated hemoglobin present in the underlying brain region are calculated using the modified Beer-Lambert Law. We depict an individual with both dark skin and curly hair (left) and an individual with light skin and straight, blond hair as two extremes of phenotypic disparity. In the left individual, both mechanical blocking due to hair texture and increased light absorption due to melanin attenuates the NIR signal, potentially leading to bias in the oxygenation estimation.

fNIRS is used to measure real-time hemodynamics in the brain and is a proxy for brain activity. Red and near-infrared light is illuminated onto the scalp by a source optode and undergoes scattering and absorption throughout the underlying brain tissue until the attenuated light is detected at another optode some distance away from the source (see Figure 1). Two phenotypic “challenges” have emerged from this. The first is in *accessing the scalp* on individuals with coarse, dense, and curly hair; present-day optodes do not ensure that light sufficiently reaches the brain when thick hair occludes the scalp. The second challenge is in acquiring quality NIRS signals once the scalp is reached. Accurate measures of hemodynamics are impacted by the light absorption and scattering properties of the layers of tissue between the scalp and the brain, namely the dermis, skull, and blood vessels, and their particular tissue chromophores, including melanin (Kharin et al., 2009; Jacques, 2013). Because darker, i.e., more melanated, skin is not accounted for in fNIRS techniques, phenotypic bias is perpetuated against darker skin, darker hair, and curlier hair as discussed below.

#### 2.1.1. Hair type

One source of bias in fNIRS is its easier usability with short, straight, thin, and lighter-colored hair. Optodes must be as flush to the scalp’s surface as possible to get an optimal signal, and securely in place. Any optical obstruction between the fiber and the scalp, especially hair, can dramatically reduce the number of photons penetrating the scalp and ultimately the surface of the brain. Conventional NIRS systems cause concern for those with coarse, curly hair because the density and thickness of the hair may obstruct the fiber and because the caps may not accommodate the larger hair volume. Even thoughtful researchers who are knowledgeable about coarse and dense hair types may struggle with maintaining participants’ optode-scalp contacts over time; for example, coarse hair tends to revert or “turn back” to its normal, unmanipulated state over time, which can move the optodes or occlude them in experiments longer than a few minutes. Or, in special populations such as children or neurodivergent people, movement and stimming may easily shift hair to suboptimal positions with respect to the optodes. Additionally, dark colored hair (of any texture) is another contributor to varying absorption properties; dark colored and thicker hair can reduce the light intensity from 20 to 50% (Koizumi et al., 1999) while light attenuation improves with lighter hair. Etienne et al. (2020) found that traditional electrodes fail to maintain low impedance on individuals with coarse, curly, and dense hair leading to exclusion of Black participants (Choy et al., 2022), so too might fNIRS optodes fail to maintain physical contact with the scalp, since they are attached in the same fashion. Even with spring-loaded grommets and tension tops on fNIRS caps, anecdotally, the signal quality for participants with coarse and/or curly hair is poor. As a result, individuals with coarse, curly, and dark hair—often people of African, African-American, and Caribbean descent—are excluded from fNIRS studies (Loussouarn et al., 2007; Takahashi, 2019; Bradford et al., 2022). Therefore, fNIRS datasets tend to underrepresent Black and Brown individuals, which supports the need for our review, as well as other individuals with this hair type. As a field we must ask: does the density, length, texture, or even the color of hair impact signal-to-noise ratio of the hemodynamics response inferred from fNIRS?

#### 2.1.2. Skin pigmentation

Another source of bias in fNIRS is its better usability with lighter skin tones. Three key underlying assumptions in using the Beer Lambert Law are that: (1) hemoglobin is the main absorber in the dermis, (2) that the tissue is optically homogeneous, and (3) that the differential pathlength is invariable across skin tones. In reality, several layers of the skin are optically heterogeneous, with melanin the dominating absorber of NIR light in the epidermis, and hemoglobin in the dermis. Functional NIRS devices assume that given a constant source-detector distance, there is a fixed light pathlength through the brain for all users. However, since melanin is a highly absorbing chromophore, higher concentrations render more absorption, thus decreasing the differential pathlength of the light, which is unaccounted for in current devices’ estimations of absolute hemoglobin. Even though fNIRS measures *relative* changes in hemoglobin, a systematic, nonlinear attenuation of the signal due to higher melanin concentrations may lead to inaccurate estimations (likely underestimations) of relative changes in oxygenation. These oversimplifying assumptions particularly bias against data from individuals with skin pigmentation darker than a two on the Fitzpatrick scale, a spectrum of skin tones ranging from 1 (lightest) to 6 (darkest).

The field has not done enough investigation into the effects of melanin on NIRS broadly. We do know that in both transmission-based NIRS such as pulse oximetry and reflectance-based NIRS like cerebral oximetry, there is evidence of larger oxygen saturation estimation error for darker skin overall and increasing error with darker pigmentation (Sun et al., 2015). Further, reflectance-based NIRS, which requires light to interact with larger bulk tissue areas, results in larger error (8% compared to transmission-based error of 2–3%) (Jubran and Tobin, 1990; Bickler et al., 2013). Simulation work for cerebral oximetry, which uses the same reflectance-based setup as fNIRS, shows that at low oxygen saturation levels—levels when patients need the most attention—the error can be up to 15% (Afshari et al., 2022). Additionally, depending on the source-detector distances, melanin might have a larger effect on data quality: larger distances would allow light to penetrate larger brain tissue volumes, decreasing the relative amount of “noise” introduced by the melanin layers in the light path. However, the influence of the amount of bulk tissue traversed has yet to be investigated with respect to skin pigmentation.

While pulse oximetry and cerebral oximetry are similar to fNIRS, one key difference is that they measure absolute hemoglobin rather than relative changes in hemoglobin. We do expect less error type in estimates of relative hemoglobin concentration changes, like those measured in continuous wave fNIRS setups. However, systematic bias in the spectroscopy technique may still exist as a function of melanin in ways that have yet to be quantified, for example, due to nonlinearities in the absorption estimations that render the relative changes in hemoglobin unreliable. Therefore, in functional NIRS, there is likely inaccuracies in calculating the hemodynamic response due to similar reasoning.

This inaccurate estimation of optically derived measures in different skin pigmentation levels is not new. The first clinically adopted NIRS device was the pulse oximeter, or pulse ox, used for non-invasive measurements of arterial oxygen saturation through the finger (Severinghaus and Honda, 1987). Developed during WWII in the racially homogeneous Japan (Millikan, 1942; Bickler and Tremper, 2022), the first pulse oximeter was adopted into clinical anesthesiology workflows in the 1980s (Severinghaus and Honda, 1987). While it has been long established that its accuracy is dependent on the calibration population (Ralston et al., 1991), its design has not been reconsidered for darker skin. Recently, COVID-19 increased hospital and home-based pulse ox monitoring (Greenhalgh et al., 2021) leading to reporting that suggest skin tone may negatively affect accuracy (Sjoding et al., 2020; Keller et al., 2022). These limitations are currently under review by the FDA (Food and Drug Administration, 2022).

### 2.2. Exploring exclusion

Methodological, experimental, and cultural limitations in current fNIRS practices contribute to what is called “convenience sampling” in brain imaging research. To accurately pinpoint convenience sampling in neuroscience research, we must assess the current phenotypic reporting practices in the theoretical and empirical neuroscience literature (Girolamo et al., 2022). In the following section, we present a literature review to determine current phenotypic and demographic reporting practices in fNIRS literature and conclude with a list of solutions to achieve an inclusive neuroscience enterprise.

## 3. Literature review

### 3.1. Methods

In May and June 2022, we conducted a literature review of demographic and phenotypic reporting from articles in top English-language Biomedical Optics and Human Neuroscience journals. The three optics and two neuroscience journals were chosen to represent a range of articles covering fNIRS hardware and algorithm development and fNIRS as a tool in basic or clinical neuroscience research, respectively. Using PubMed, we saved a catalog of all articles in the given time range, selected journal name, and the keyword “fNIRS.” For the biomedical optics articles, we selected a 15-year time range; for the human neuroscience articles, we selected a 5-year time range. This time difference is because fNIRS’ adoption into basic research has understandably lagged fNIRS development; in all, both time ranges include the present day. Articles were retrieved on the open web or via subscription at the authors’ institution. For each article, we documented the number of participants, country of testing, any quantitative or qualitative reports of data exclusion, and participant demographics including: mention of sex or gender; mention of race, ethnicity, or nationality; mention of melanin, pigmentation, or Fitzpatrick scale; and mention of hair type. Animal and *in silico* studies were reported as “N/A.” We intentionally included sex/gender reporting in the analyses to compare as a baseline exemplar of “good” demographic reporting, since the NIH and many publishing bodies have encouraged or mandated reporting increasingly in the past 3 decades (National Academies of Sciences, Engineering, and Medicine, 2022).

## 4. Results

**Figure 2:**
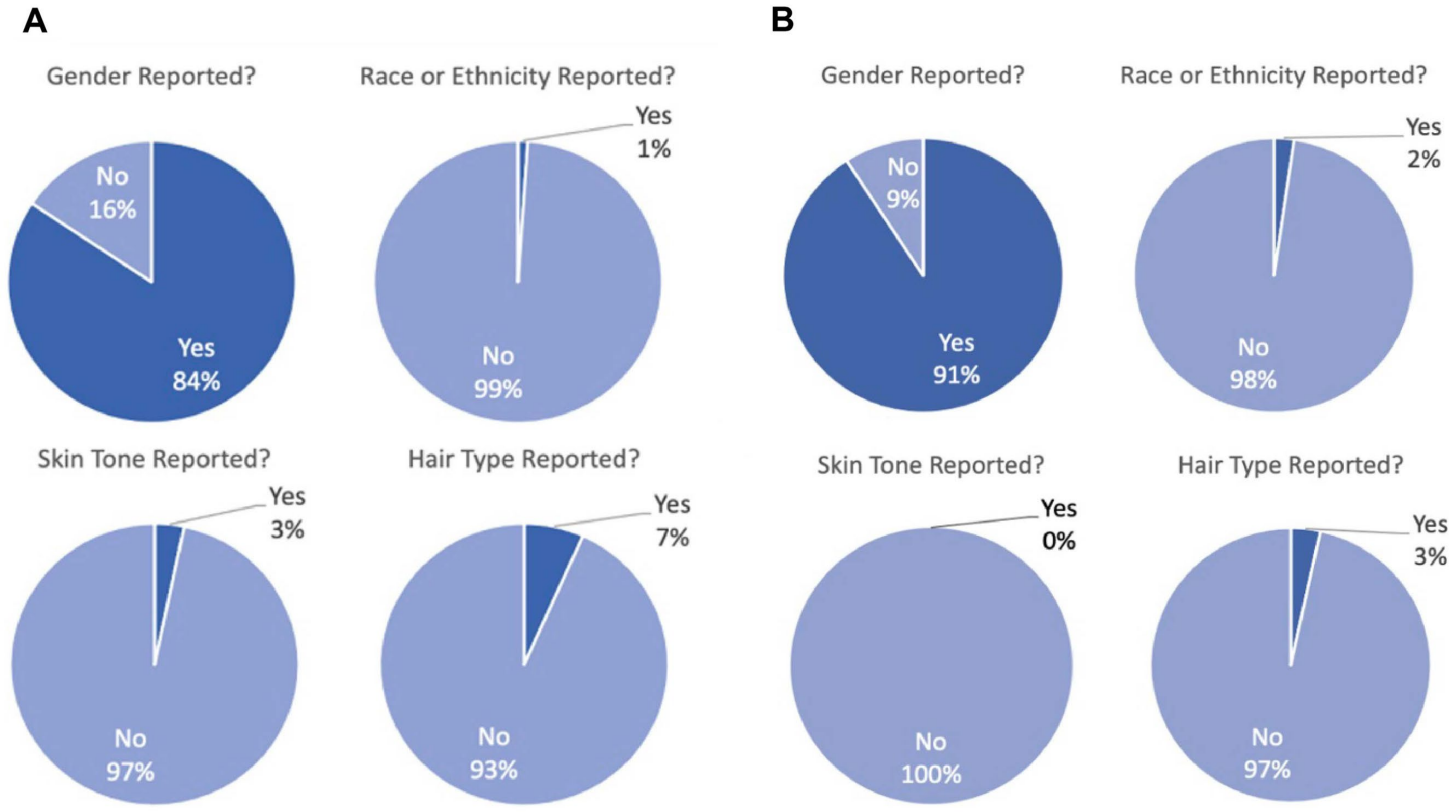
**(A)** Demographic reporting for 90 articles with empirical human fNIRS data in three top biomedical optics journals. Overwhelmingly, gender is reported (“yes”) whereas race/ethnicity, skin pigmentation, and hair type are overwhelmingly not reported (“no”). **(B)** Demographic reporting for 87 articles with empirical human fNIRS data in two top human neuroscience journals. Overwhelmingly, gender is reported (“yes”) whereas race/ethnicity, skin pigmentation, and hair type are overwhelmingly not reported (“no”).

From three top optics journals, we identified 110 articles from 2007 to 2022. We excluded *in silico* studies or those using animals, leaving a total of 90 articles with human volunteer participants (Figure 2A). While most studies reported gender (84.4%) as we predicted, nearly all articles failed to report phenotypic characteristics about participants being race/ethnicity (98.9%), skin pigmentation (96.7%), or hair type (93.3%). Over time, this trend does not seem to be improving (see Supplementary Figure 1).

We then repeated this analysis for two top human neuroscience journals that together publish a large proportion of basic science fNIRS articles. We identified 87 papers from 2017 to 2022 that used fNIRS as a tool (Figure 2B). Again, the vast majority of studies report gender (90.8%), but do not report race/ethnicity (97.7%), skin pigmentation (100%), or hair type (96.6%).

Lastly, we looked at the types of exclusion that were reported from all five journals. Only 69 of the 177 total articles (39.0%) mentioned if any participants were excluded *for any reason*. Of these 69, eight (11.6%) explicitly mention hair and four (5.80%) cite it as the main reason for the exclusion or withdrawal. The majority of the articles shared general reasons for dismissing a participant like “noisy data across channels,” “poor light shielding,” “technical issues… or low quality fNIRS data…,” “Bad fNIRS signal and technical issues,” and “poor cap fit.” The four articles cited thick or dark hair as being the reason for why a participant may have been excluded saying “poor data quality resulting from the subject’s relatively thick, black hair,” “unable to collect effective signals from fNIRS due to the participant’s thick, strong hair,” “had a lot of hair to obstruct light,” and “presumably due to dense and/or dark-colored hair.” No articles mention skin tone as being the primary source of signal noise. Race/ethnicity was the second least reported demographic and was typically reported by country of origin (e.g., “All participants were Chinese.”). A list of all the reasons for exclusion from the 69 articles are provided in Supplementary Table 1.

Unfortunately, because of the low level of demographic reporting, we were not able to present data comparing the relative exclusion of marginalized and majority phenotypes.

## 5. Discussion

Our results point to two distinct issues: the under-reporting of exclusion and the potential, but unconfirmed, disproportionate exclusion of marginalized phenotypes. While recruiting diverse participants can prove challenging, simply reporting the participant makeup should be straightforward (see section 5 for more discussion and recommendations). It is hard to disentangle the contributing factors toward exclusion of marginalized groups in neuroimaging: there is phenotypic bias, but also less access, lower interest and response rates (due to perceived racial bias), claims that data is “unusable,” and health disparities (Rad et al., 2018; Louis et al., 2022; Webb et al., 2022; Ricard et al., 2023). There is a long literature about these issues, especially medical mistrust among African-Americans, as well as how to alleviate these issues (see introduction in Otado et al., 2015). However, when comparing to the representation of Black/African-American identifying individuals in the United states (13%) and that of NIH-funded neuroimaging studies generally (7%), the anecdotal indications that there are not nearly *any* Black participants in fNIRS is alarming and points to phenotypic bias contributions beyond the typical exclusion factors that lead to underrepresentation of Black participants (NIH stats from public access database).

In surveying biomedical optics journals, we sought to target work by the engineers who design fNIRS systems, those responsible for inclusive design practices. In surveying human neuroscience journals, we targeted work by end users. In both pursuits, we found that gender was reported in the vast majority of articles. This is likely due to the widespread adoption of gender reporting from NIH mandates that touched the animal research world as well as human research (and to our knowledge, other animals do not observe the social construct of race).

While four articles did report hair type and should be commended, there was only one article that explicitly mentioned hair type, hair color, *and* Fitzpatrick skin color. We especially commend that group for being transparent about the influences on their results and believe it should be the standard.

## 6. Recommendations

In the absence of adequate reporting of demographic data to determine exclusion trends in fNIRS literature, we think its important to highlight ways that the community—both developers and practitioners—can be more inclusive and more upfront about the inclusivity of their data. Based on our results, there is unequivocally exclusion based on, at minimum, the curliness and darkness of hair. To address this embedded bias, fNIRS tools and practices must change to accurately represent a heterogeneous population. The transition of fNIRS technology to more inclusive methodologies will require concerted efforts from engineers, scientists, clinicians, and imaging professionals following the example of groups already developing creative solutions.

### 6.1. Engineering solutions

Some groups are actively addressing phenotypic bias limitations of fNIRS while maintaining other design requirements such as direct and prolonged contact with the scalp, maintenance of good signal-to-noise ratios, and increasingly higher spatial resolutions:

- A Texas-based group designed brush-type optodes to improve photon transmission and demonstrated its applicability with dark hair colors and high hair density by estimating power attenuation through a derived analytical model (Khan et al., 2012).
- More recently, a team led by Sossena Wood began developing both novel inclusive optodes for curly hair and better algorithms to account for skin pigmentation (see award announcement here). The novel optode adapters anchor onto the scalp using the strength of strategically placed braids and improve the optical contact onto the scalp compared to commercially available flat optodes. An alternative to strategic braiding is to have the hair pre-braided before the visit into very small braids (which allow for more scalp contact options) or to have the hair fully washed, detangled, and dried while in a “stretched” state (via a loose ponytail, braids, twists, etc.). To achieve these specifications, volunteers must be given reasonably advanced notice, just as fMRI volunteers are given notice about piercings that need healing or larger hairstyles that might not fit into head coils. The team, which includes some authors from the aforementioned EEG work (Etienne et al., 2020), has recently expanded to create novel pulse ox as well.
- A few studies mention personalized approaches to inclusive fNIRS setup, especially cap interfacing and design, a critical element to achieve quality optical contact. Sun et al. mounts light sources and detectors on a custom silicone cap to maintain contact (see Supplementary Figure 2; Sun et al., 2022). The same group at the University of Michigan uses crochet hooks with LED lights to gently move hair during the optimization process before inserting optodes. While cap customization improves optode contact for different hair lengths and some hair types, the design may not be universal. For example, using crochet hooks can be painful for black hair as it tangles, and research assistants must be trained to do it.

### 6.2. Best inclusive practices for fNIRS researchers

There are feasible approaches that researchers may consider to curb phenotypic exclusion and increase equity in the field. We also point to other work specific to recruitment practices outlined in our supplement section 1 (Dancy et al., 2004; Auksztulewicz and Friston, 2015; Habibi et al., 2015; Otado et al., 2015; Garavan et al., 2018; Wieland et al., 2021).

#### 6.2.1. Report demographics and phenotypes

We commend the one group in our sample that provided all demographic information upfront as well as the other groups that were honest about their exclusion of thick and coarse hair. Researchers involved in neuroimaging should explicitly report the racial and gender breakdown of their sample and, especially when there is exclusion of certain participants, describe the phenotypes such as hair color, hair type, and skin tone (Yücel et al., 2021). Data about hair type and skin tone can be surveyed or judged by an experimenter familiar with the Fitzpatrick scales and hair typing scales such as the Andre Walker System or the L’Oreal system (Loussouarn et al., 2007). Researchers should also consider the benefit of systematically quantifying the association of hair type, density, and melanin content of the scalp with fNIRS measurements. Formally defining these limitations through a systematic review will enable engineers to approach future advancements driven by these factors.

#### 6.2.2. Adopt inclusive methodologies and hire a diverse research team

Although fNIRS systems need improvements, there are other reasons why darker skinned and curlier haired individuals are excluded from psychological research and design solutions. Many standard procedures foster an unpleasant environment and result in voluntary participant withdrawal from marginalized backgrounds especially for special populations in which fNIRS is beneficial. For example, children with darker pigmented skin and curlier hair textures (and their parents) may get frustrated and lose trust in the researchers because of the complex setup process, which involves repeatedly moving the cap and hair. Moreover, individuals with intellectual disabilities—a large proportion due to fNIRS’ portability and motion tolerance—may not be able to handle the inconvenience.

To improve participant experience, researchers should train to work with a range of hair types as standard practice. Adverse outcomes of unpreparedness include longer setup times, microaggressions, participant discomfort, and participant dropout. fNIRS researchers should consider developing guidelines for preparation that will serve as standard operating procedure. Given some similarities in configuration and setup between EEG systems and fNIRS equipment, following Etienne et al.’s suggestion for adopting braiding techniques to separate hair might be a good solution. For higher spatial resolution setups, labs can consider application of (or development of) suggestions as outlined in *A Guide to Hair Preparation for EEG Studies,* available online (Richardson et al., 2021).

Aside from building trust with marginalized communities, hiring and training a research team with diversity in mind can bring in practitioners who can effectively relate to marginalized participants before, during, and after laboratory visits. With better familiarity of marginalized communities, researchers can identify and prevent barriers to participation, making their studies more accessible. Similarly, allocating grant money to hire a hair consultant while considering custom setups is ideal.

### 6.3. IRBs, journals, governing, and foundations: mandated reporting

The responsibility of race and gender reporting does not simply fall on individual researchers, but also on the funding, publishing, and ethics bodies to which they are beholden. Each of these entities have a responsibility to mandate reporting of demographics and question any researchers who include race- or phenotype-based exclusion criteria in their studies. As highlighted in Webb et al. (2022), IRBs are in place to ensure that institutional research is both rigorous and ethical. IRB personnel should receive ongoing training on the presence of racial bias in research devices and offer institutionally mandated inclusive best practices to researchers.

Similarly, funding bodies and journals should require demographic reporting and data demographic disaggregation. Foundations should invite research proposals explicitly asking the questions of the present article: who is being excluded and why, both technologically and culturally? Finally, foundations should fund innovative and equitable technologies, like the work of the team led by Sossena Wood at Carnegie Mellon University and the team led by Meryem Yücel at Boston University, both funded by Meta Reality Labs.

Pressure for change will mount with the help of concerted action-based efforts. More scientific organizations and foundations should provide support for neuroscientists and engineers via resources like the Neuroethics Framework formed by IEEE. At the Federal level, passing the Diverse and Equitable Participation in Clinical Trials (DEPICT) Act and similar legislature would help, provide the FDA with the authority to require diverse representation in clinical trials.

Though the onus of progress is collective, the authors herein embolden the entire fNIRS community to assume individual responsibility for conducting inclusive work within their own realms of influence, including as researchers, journal editors, manuscript and grant reviewers, IRB members, and leaders in their own scientific and social circles.

## Data availability statement

The raw data supporting the conclusions of this article will be made available by the authors, without undue reservation.

## Author contributions

JK and KK: conceptualization. JK and HP: methodology, data curation, and visualization. JK, HP, TP, and NN: investigation. JK, HP, KK, TP, and NN: writing (original draft). JK and SW: writing (review and editing) and supervision. SW: funding acquisition. All authors contributed to the article and approved the submitted version.

## Funding

JK was supported by NIH K00NS115331 and the Burroughs Wellcome Fund Postdoctoral Diversity Enrichment Program. TP was supported by NSF GRFP #1752134. HP was supported by NIH award T32CA079443. Funding for this investigation was provided by a Meta Reality Labs grant to SW and JK.

## Conflict of interest

JK was employed by Precision Neuroscopics, Inc., a designer of inclusive EEG electrodes.

This study received funding from Meta Reality Labs. The funder was not involved in the study design, collection, analysis, interpretation of data, the writing of this article or the decision to submit it for publication. All authors declare no other competinginterests.

## Publisher’s note

All claims expressed in this article are solely those of the authors and do not necessarily represent those of their affiliated organizations, or those of the publisher, the editors and the reviewers. Any product that may be evaluated in this article, or claim that may be made by its manufacturer, is not guaranteed or endorsed by the publisher.

## Supplementary material

The Supplementary material for this article can be found online at: https://www.frontiersin.org/articles/10.3389/fnins.2023.1086208/full#supplementary-material
