# Corrigendum: Demographic reporting and phenotypic exclusion in fNIRS

**DOI:** 10.3389/fnins.2023.1331375

**Published:** 2023-12-01

**Authors:** Jasmine Kwasa, Hannah M. Peterson, Kavon Karrobi, Lietsel Jones, Termara Parker, Nia Nickerson, Sossena Wood

**Affiliations:** ^1^Neuroscience Institute, Carnegie Mellon University, Pittsburgh, PA, United States; ^2^Center for Systems Biology, Massachusetts General Hospital, Boston, MA, United States; ^3^Department of Biomedical Engineering, Boston University, Boston, MA, United States; ^4^Burnett School of Biomedical Sciences, University of Central Florida, Orlando, FL, United States; ^5^Interdepartmental Neuroscience Program, School of Medicine, Yale University, New Haven, CT, United States; ^6^Combined Program in Education and Psychology, University of Michigan, Ann Arbor, MI, United States; ^7^Department of Biomedical Engineering, Carnegie Mellon University, Pittsburgh, PA, United States

**Keywords:** fNIRS (functional near-infrared spectroscopy), inclusion, neuroimaging, melanin, biomedical optics

In the published article, there was an error in the Funding statement.

“JK was supported by NIH K00NS115331. TP was supported by NSF GRFP #1752134. HP was supported by NIH award T32CA079443. Funding for this investigation was provided by a Meta Reality Labs grant to SW and JK”. The correct Funding statement appears below.

